# A comprehensive guide to miniaturized percutaneous nephrolithotomy: International Alliance of Urolithiasis (IAU) consensus on best practices

**DOI:** 10.1186/s40779-025-00602-6

**Published:** 2025-03-24

**Authors:** Kesavapillai Subramonian

**Affiliations:** Department of Urology, Queen Elizabeth Hospital Birmingham, Birmingham, B15 2GW UK

**Keywords:** Miniaturized percutaneous nephrolithotomy (mPCNL), International Alliance of Urolithiasis (IAU) consensus, Delphi process, Kidney stone surgery

The International Alliance of Urolithiasis (IAU) consensus on miniaturized percutaneous nephrolithotomy (mPCNL) [1] is produced by an experienced international panel of experts in kidney stone surgery and is based on a systematic review of literature and a Delphi process, ensuring that recommendations are grounded in evidence.

The consensus provides clear and practical guidelines for various aspects of mPCNL, such as tract size, lithotripsy techniques, postoperative management, and so on. There is a high level of consensus in excess of 90% for a number statements including those on less trauma caused by the procedure, computed tomography (CT) as the imaging modality of choice for preoperative imaging, choice of general anaesthesia, prone or supine positioning, choice of fluoroscopic imaging for access, urologists gaining PCNL access and CT scan for follow up imaging to check clearance.

Key strengths of the consensus include comprehensive coverage of all aspects related to mPCNL, evidence based approach, and clarity of advice regarding when and how to perform mPCNL. However, there are a few areas where the consensus could be strengthened. While the consensus attempts to clarify the definition of mPCNL using tract size below 18 Fr, there is still some ambiguity in the terminology used especially when considering tract sizes below 14 Fr. Although the terminology “mPCNL” is used in the article as all-encompassing for all tract sizes below 18 Fr, most of the guidance statement is applicable to tract sizes of 12−18 Fr. A more standardized classification could help to improve communication and understanding among urologists [2]. While the consensus discusses the benefits of mPCNL in terms of reduced bleeding, pain, and hospital stay, it could be further strengthened by including more data on patient-reported outcomes, such as quality of life and patient satisfaction.

Another potential enhancement involves incorporating cost-effectiveness analyses. Given the varying costs associated with the procedure and the potential long-term savings from reduced complications and hospital stays, data on cost-effectiveness would offer urologists a more complete perspective on mPCNL’s practical implications [3]. Additionally, aligning the procedure with the green agenda by assessing its environmental impact would contribute to the growing focus on sustainability in healthcare [4].

In conclusion, the IAU consensus on mPCNL provides a comprehensive framework for urologists seeking to implement this technique in their practice. The consensus covers key areas such as indications, preoperative workup, procedural tips, and postoperative care. While the consensus provides clear guidelines for various aspects of mPCNL, including tract size, preoperative preparation, lithotripsy techniques, exit strategy, and postoperative management, there is still scope for improvement by including more data on patient-reported outcomes, cost-effectiveness and addressing emerging technologies and green agenda.

## Data Availability

Not applicable.

## Abbreviations

mPCNL: Miniaturized percutaneous nephrolithotomy
IAU: International Alliance of Urolithiasis
